# Intravenous Iron Therapy in Patients with Iron Deficiency Anemia: Dosing Considerations

**DOI:** 10.1155/2015/763576

**Published:** 2015-07-15

**Authors:** Todd A. Koch, Jennifer Myers, Lawrence Tim Goodnough

**Affiliations:** ^1^Luitpold Pharmaceuticals, Inc., Norristown, PA 19403, USA; ^2^St. John's University, Jamaica, NY 11439, USA; ^3^Department of Pathology and Medicine (Hematology), Stanford, CA 94305, USA

## Abstract

*Objective.* To provide clinicians with evidence-based guidance for iron therapy dosing in patients with iron deficiency anemia (IDA), we conducted a study examining the benefits of a higher cumulative dose of intravenous (IV) iron than what is typically administered. *Methods.* We first individually analyzed 5 clinical studies, averaging the total iron deficit across all patients utilizing a modified Ganzoni formula; we then similarly analyzed 2 larger clinical studies. For the second of the larger studies (Study 7), we also compared the efficacy and retreatment requirements of a cumulative dose of 1500 mg ferric carboxymaltose (FCM) to 1000 mg iron sucrose (IS). *Results.* The average iron deficit was calculated to be 1531 mg for patients in Studies 1–5 and 1392 mg for patients in Studies 6-7. The percentage of patients who were *retreated* with IV iron between Days 56 and 90 was significantly (*p* < 0.001) lower (5.6%) in the 1500 mg group, compared to the 1000 mg group (11.1%). *Conclusions.* Our data suggests that a total cumulative dose of 1000 mg of IV iron may be insufficient for iron repletion in a majority of patients with IDA and a dose of 1500 mg is closer to the actual iron deficit in these patients.

## 1. Introduction

Iron is an essential element and its balance must be maintained for proper physiologic functioning. Blood loss, a major cause of iron deficiency, is highly prevalent (e.g., females with menses and patients with chronic occult gastrointestinal (GI) blood loss) and requires proper diagnosis and management [1–4]. Therapeutic management of IDA is focused primarily on repletion of iron stores [1–4]. While iron deficient individuals without inflammation may respond to oral iron therapy, administration of IV iron is beneficial in many patient populations, including those with inflammation (resulting, e.g., from kidney disease, heart failure, or rheumatological diseases), patients who cannot tolerate oral iron, and patients who are noncompliant with oral iron therapy [5–8]. Even under the best of circumstances, oral iron is not well tolerated, and patients are often nonadherent for a variety of reasons, including intolerable side effects and the need for multiple daily doses [9]. The frequently poor absorption of oral iron, moreover, can contribute to suboptimal patient response.

The hepcidin response in anemic patients having inflammatory conditions, such as inflammatory bowel disease (IBD), inhibits GI absorption of oral iron [10]. Moreover, hepcidin impacts iron homeostasis in patients with concurrent inflammation (e.g., repressed recycling of iron from the reticuloendothelial system and sequestration in bone marrow); this may limit both oral and IV iron supplementation and may serve to explain why such patients remain iron deficient despite multiple courses of therapy [6, 10, 11].

Cancer-related anemia (CRA) has multiple etiologies, including chemotherapy-induced myelosuppression, blood loss, functional iron deficiency, erythropoietin deficiency due to renal disease, and marrow involvement with tumor, among others. The most common treatment options for CRA include iron therapy, erythropoietic-stimulating agents (ESAs), and red cell transfusion. Safety concerns as well as restrictions and reimbursement issues surrounding ESA therapy for CRA have resulted in suboptimal treatment. Many believe that more routine use of IV iron for CRA and chemotherapy-induced anemia (CIA) is appropriate in view of existing evidence. Oncology patients whose CIA is treated with ESAs, furthermore, respond better to IV iron therapy than to oral supplementation [7, 13–19].

Table 1 illustrates various conditions where IV iron therapy may be warranted.

Despite beneficial effects in a wide range of patients, administration of IV iron may generate oxidative stress and other inflammatory changes, and the risk-benefit profile of IV iron continues to undergo evaluation in renal dialysis patients [20, 21], as well as patients with anemia due to other chronic diseases [22]. The long-term effects of IV iron preparations will require further study in relevant clinical settings, [23] as will the long-term deleterious effects of allogeneic blood transfusions [24–26].

IV iron preparations currently approved in the US are listed in Table 2 [8, 10, 27–36]. Beginning with the first iron dextran product introduced, the recommended cumulative replacement dose for many of these products has been approximately 1000 mg of iron [29–35].

A patient's total body iron deficit can be calculated using the Ganzoni formula (total iron dose = [actual body weight × (15-actual Hb)] × 2.4 + iron stores) [32]. Because many view this formula as inconvenient, it is not consistently used in clinical practice [37]. Although use of the Ganzoni formula is ideally the best way to select dose, it is impractical, partly because product labels state specific dosing regimens. In typical clinical practice, doses are more efficiently chosen based on approved product labels and local protocols, and only in the Dexferrum (iron dextran injection, USP) and INFeD (iron dextran injection, USP) prescribing information is a weight and Hb-based table available to calculate a patient's total iron requirement utilizing similar formula. There are also only a limited number of clinical practice guidelines regarding the use of a total cumulative repletion dose of IV iron in IDA patients, and, as mentioned above, the FDA-approved labeling for many IV iron products recommends a total cumulative dose of approximately 1000 mg. Currently, there is no consensus regarding the most appropriate iron deficit repletion dosing in patients with IDA, partly because the iron dosing selected for virtually all trials has been based largely on clinical judgment, clinical guidelines in nephrology, or best estimates from past results. In this retrospective study, we systematically explored the iron deficit in patients who received IV iron in clinical studies and examined the potential benefits (i.e., normalization of Hb and time to retreatment with IV iron) of a higher cumulative dose of IV iron than what is typically administered, with the goal of providing clinicians with practical, evidence-based guidance for determining iron dosing requirements in a wide range of patients with IDA.

## 2. Materials and Methods

In this study, we used the same population recruited from previous clinical trials [38–44]. These studies adhered to US federal regulations and were performed in accordance with the Declaration of Helsinki and lastly protocols and informed consent forms were approved by local or national institutional review boards. All participants in these studies provided written informed consent. Patient records/information were anonymized and deidentified prior to analysis.

In Studies 1–5 (summarized below), each patient's iron deficit (mg) had been originally calculated and dose of iron administered, according to a modified Ganzoni formula: subject weight in kg × [15-current Hb g/dL] × 2.4 + 500, as specified in each study protocol. The Ganzoni formula had been modified for use in these studies to help alleviate any potential for iron overload in subjects who had a transferrin saturation (TSAT) >20% and ferritin >50 ng/mL at study entry. For these subjects, a conservative estimate was made, and the additional 500 mg from the formula to replete iron stores was not added to the total iron requirement. Each study administered IV iron (ferric carboxymaltose, FCM) as a total cumulative dose to randomized patients based upon the iron deficit so calculated. In analyzing each study, we utilized the baseline iron deficits for each patient using the same method and then averaged the total iron deficit across patients. These clinical studies examined IDA in postpartum patients, patients with heavy uterine bleeding (HUB), non-dialysis-dependent chronic kidney disease (NDD-CKD), GI disorders, and other underlying conditions.

Following are short descriptions of each study:

1. Comparison of the safety and efficacy of IV iron (FCM) and oral iron (ferrous sulfate) in patients with postpartum anemia (*N* = 361) [38], NCT00396292.
2. Comparison of the safety and efficacy of IV iron (FCM) and oral iron (ferrous sulfate) in the treatment of IDA secondary to HUB (*N* = 477) [39], NCT00395993.
3. Comparison of the safety and efficacy of IV iron (FCM) and oral iron (ferrous sulfate) in the treatment of postpartum patients (*N* = 291) [40], NCT00354484.
4. Comparison of the safety and tolerability of IV iron (FCM) and standard medical care (oral and IV iron) in treating IDA of various etiologies (*N* = 708) [41], NCT00703937.
5. Comparison of the safety and tolerability of IV iron (FCM) and iron dextran in treating IDA of various etiologies (*N* = 160) [42], NCT00704028.

Following review of Studies 1–5, two larger studies (6 and 7) that utilized 1500 mg IV iron (as specified in the protocols) were examined. Although the modified Ganzoni formula was* not* specified in the protocols to determine dose requirements in these 2 studies, we did apply the formula to determine each patient's baseline iron deficit in a separate retrospective* post hoc* analysis of each study. We then averaged the total iron deficit across patients. Additionally, Study 7 compared the safety and efficacy of 1500 mg of IV iron (as FCM) to 1000 mg of IV iron (as iron sucrose [IS]) examining any potential efficacy or safety difference between the two dosing regimens.

A short summary of Studies 6 and 7 follows:

(6) Comparison of 1500 mg IV iron (FCM) with oral iron and IV iron standard of care (SoC) therapy (as determined by the investigator) in patients with IDA of various etiologies who had an unsatisfactory response to oral iron or were deemed inappropriate for oral iron [43], NCT00982007.
(7) Comparison of the safety and efficacy of 1500 mg (FCM) to 1000 mg of IV iron (IS) in patients with IDA and NDD-CKD [44], NCT00981045.

*Statistical Analysis.* Baseline iron deficits in each clinical study were calculated for all subjects who were randomized to receive IV iron. In Study 5 [42], iron deficits were calculated for all subjects, as the comparator (iron dextran) was also dosed based on the modified Ganzoni formula and was summarized with descriptive statistics. For the iron deficit calculations performed for Studies 6 and 7, all subjects in the Safety Population were included. The iron deficits were averaged and the standard deviation was generated.

For Study 7, the Safety Population consisted of all subjects who received a dose of randomized treatment. The intent-to-treat (ITT) population for evaluating all efficacy endpoints consisted of all subjects from the Safety Population who received at least 1 dose of randomized study medication and had at least 1 postbaseline Hb assessment. Treatment assignments were analyzed according to the actual treatment received. The differences between 1500 mg and 1000 mg for time-to-event variables in Study 7 were assessed with the point estimate and 95% CI for the hazard ratio calculated from a Cox proportional hazards model. Treatment group differences were assessed using the Cox proportional hazards model with treatment as a fixed factor. In addition, *p* values for treatment differences were provided from the log-rank test. Time-to-event variables are displayed descriptively as Kaplan-Meier curves.

All statistical tests were* post hoc* with no adjustment to type I error for multiple comparisons.

## 3. Results

The average total iron deficits for patients in the 7 cited trials are summarized in Tables 3 and 4. The overall average total iron deficit in the initial 5 clinical trials was 1531 mg (Table 3). Total iron requirements among patients in each cohort in Studies 6 and 7 are summarized in Table 4. In Study 6, the average calculated iron deficit (Cohorts 1 and 2) was 1496 mg. In Study 7, the average calculated iron deficit for patients receiving either 1500 mg or 1000 mg was 1352 mg. Overall, the average total iron deficit for clinical Studies 6 and 7 was 1392 mg.

In Study 7, study participants were randomized to receive either two 750 mg doses of IV iron (FCM) 7 days apart or IS 200 mg administered in up to 5 infusions over 14 days. The primary efficacy endpoint was the mean change in Hb from baseline to highest reported Hb (from baseline to Day 56). Patients were followed up for safety to Day 120. The mean total dose of iron received was 1464 mg in the 1500 mg group and 963 mg in the 1000 mg group. Mean baseline Hb values were 10.31 g/dL for the 1500 mg group and 10.32 g/dL for the 1000 mg group.

In this study, the mean increase in Hb overall was 1.13 g/dL in the 1500 mg group and 0.92 g/dL in the 1000 mg group (95% CI, 0.13–0.28), meeting the prespecified endpoint of noninferiority of 1500 mg to 1000 mg. Additionally, as evidenced by the 95% CI not including 0, 1500 mg was superior to 1000 mg in increasing Hb.

**Figure 1:**
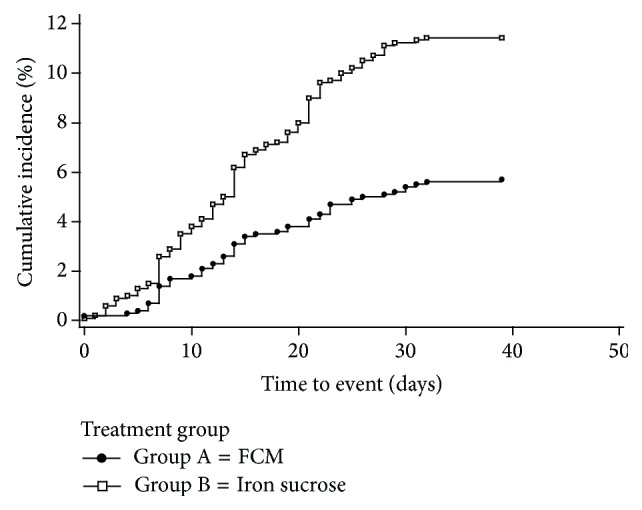
The time to additional intravenous (IV) iron after Day 56 comparing 1500 mg to 1000 mg IV iron in the Safety Population of Study 7. Data on file, Luitpold Pharmaceuticals, Inc.

The proportion of patients in the 1500 mg group who were* retreated* with IV iron between Days 56 and 90 (Safety Population) was significantly (*p* < 0.001) lower, 71/1276 (5.6%), than the 142/1285 (11.1%) patients who required retreatment in the 1000 mg group (Table 5).  Figure 1 displays the time from Day 56 to additional IV iron when comparing 1500 mg to 1000 mg.

*Post hoc* analyses of patients with Hb >12 g/dL at end of treatment (Day 56) and time to first Hb >11 g/dL and >12 g/dL and Hb increase ≥1 g/dL were conducted. The proportion of patients with Hb >12 g/dL at the end of treatment (baseline to Day 56) was 265/1249 in the 1500 mg group (24.4%) and 169/1244 in the 1000 mg group (15.6%), *p* = 0.001 (Table 6).

Patients who received 1500 mg IV iron were also more likely to achieve Hb >11 g/dL, Hb >12 g/dL, or an increase in Hb ≥1 g/dL compared with those receiving 1000 mg (Table 7).

**Figure 2:**
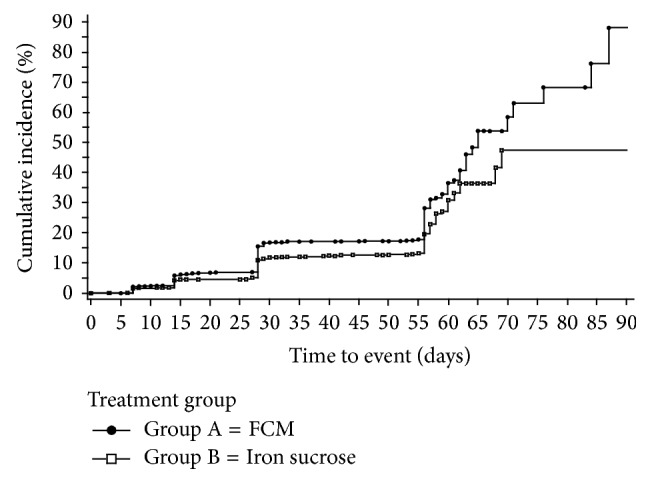
The time from randomization to first hemoglobin >12 g/dL in patients who received 1500 mg IV iron and patients who received 1000 mg from Study 7, *p* < 0.001. Day 56 is the last study visit, and at the discretion of the investigator, patients were allowed to be retreated with additional IV iron between Days 56 and 90. Data on file, Luitpold Pharmaceuticals, Inc.

Furthermore, the times to first Hb >11 g/dL and >12 g/dL and to Hb increases ≥1 g/dL were all statistically significantly shorter for the 1500 mg group than for the 1000 mg group (*p* = 0.013, *p* < 0.001, and *p* < 0.001, resp.).  Figure 2 presents the Kaplan-Meier analysis for time to first Hb >12 g/dL.

The 1500 mg total cumulative dose had a similar safety profile to that of 1000 mg of IS, demonstrating that 50% more iron in the form of FCM can be administered while maintaining a safety profile comparable to that of IS [44].

## 4. Discussion

In the US, it has become common practice to administer a cumulative dose of approximately 1000 mg of IV iron (in divided doses) for the treatment of IDA. This is due, in large part, to the use of IV iron in nephrology. Both the Kidney Disease Outcomes Quality Initiative (KDOQI), initially developed in 1996, and the more recent Kidney Disease: Improving Global Outcomes (KDIGO) practice guidelines provide recommendations for the treatment of IDA utilizing IV iron. In the randomized controlled trials reviewed to develop the guidelines, a cumulative dose of 1000 mg of IV iron was utilized [45, 46]. Although that has now become the standard therapeutic dose for iron deficiency of various etiologies in light of a wealth of safety and efficacy data, it may not provide repletion of iron that is sufficient to alleviate the iron deficient state, thereby necessitating retreatment or creating the potential for a subtherapeutic response.

Despite these recommendations, in many clinical situations the treatment of IDA with IV iron has not been limited to a cumulative dose of 1000 mg. In oncology patients, for example, the National Comprehensive Cancer Network (NCCN) states that if the calculated dose exceeds 1000 mg, the remaining dose may be given after 4 weeks if the Hb response is inadequate [47].

Additionally, in two randomized controlled trials involving IV iron supplementation in oncology patients, a total of up to 3000 mg iron was administered in weekly doses of 100 mg [48]. In another prospective, randomized, controlled trial, patients with chemotherapy-related anemia received cumulative doses of IV iron ranging from 1000 to 3000 mg [7].

Guidelines for the management of IDA in inflammatory bowel disease (IBD), moreover, recommend IV iron as the preferred route of administration and state that anemic IBD patients rarely present with total iron deficits below 1000 mg. These guidelines recommend use of the Ganzoni formula to estimate iron replacement needs, and in controlled trials, up to 3600 mg of iron sucrose has been administered safely (up to TSAT >50%) [49]. A 2011 review by Gozzard [50] further highlights numerous clinical situations requiring doses of IV iron above a cumulative dose of 1000 mg. Congruent with evidence reported in the international IBD guidelines, the article states that cumulative doses up to 3600 mg of IV iron may be administered safely in these patients. The review also suggests that higher doses of IV iron may overcome impaired iron absorption associated with hepcidin blockade in this patient population. In another multiple-dose, phase II/III study of IDA patients with GI disorders, mean total cumulative doses of 1800 mg IV iron were administered [51]. Clinical evidence also indicates that iron requirements of 1000 to 1500 mg or higher may be required in patients with NDD-CKD to attain target ferritin and Hb levels, up to 1600 mg may be required in obstetric patients, and as much as 2000 mg may be needed in patients with heavy or abnormal menstrual bleeding [50].

To help determine the optimal means of administering these higher doses, it is important to note that the degradation kinetics, and therefore the safety, of parenteral iron products are directly related to the molecular weight and stability of the iron complex [52–56].

Complexes can be generally classified as labile or robust (kinetic variability, i.e., how fast the ligands coordinated to the iron can be exchanged) and weak or strong (thermodynamic variability, i.e., how strongly the ligands are bound to the iron and thus how much energy is required to dissociate a ligand from the iron) or any intermediate state [52]. The reactivity of each complex correlates inversely with its molecular weight; larger complexes are less prone to release significant amounts of labile iron or react directly with transferrin [53, 54]. Type I complexes such as iron dextran preparations (INFeD Dexferrum) or FCM (Injectafer) have a high molecular weight and a high structural homogeneity and thereby deliver iron from the complex to transferrin in a regulated way via macrophage endocytosis and subsequent controlled export [52, 55]. They also bind iron tightly as nonionic polynuclear iron(III) hydroxide and do not release large amounts of iron ions into the blood. Such complexes can be administered intravenously and are clinically well tolerated even when administered at high doses. For less stable iron complexes, the maximum single doses are significantly lower and the administration times are drastically longer [54, 56].

FCM is a stable type I polynuclear iron(III) hydroxide carbohydrate complex that prevents the partial release of iron to serum ferritin observed with IS, allowing administration of high doses, since this iron is available only via reticuloendothelial processing [37, 57, 58]. FCM can be administered as a single 750 mg dose via a slow IV push injection over 7.5 minutes or as an IV infusion over at least 15 minutes. The second dose is administered at least 7 days later for a recommended cumulative dose of 1500 mg iron [36]. Use of high doses reduces the number of infusions, enabling the possibility of cost reductions compared to multiple administrations [59–62].

In our study, a modified Ganzoni formula was used to calculate total iron deficits in patients from 5 clinical studies involving FCM. After analyzing each study individually, we found the overall average iron deficit in those trials to be 1531 mg, suggesting that patients having IDA of various etiologies may benefit from a higher cumulative dose of IV iron than what is typically administered in clinical practice utilizing most of the currently available IV iron formulations.

Using the same modified Ganzoni formula, total iron deficits were also calculated in our* post hoc* analyses of the 2 larger studies (6 and 7) involving patients with IDA secondary to numerous underlying disorders, including HUB, GI diseases, and CKD. In Study 6, the average calculated iron deficit was 1496 mg. In Study 7, the average calculated iron deficit for patients receiving either 1500 mg IV iron, as FCM, or 1000 mg of IS was 1352 mg. The lower figure may be due to higher baseline ferritin and TSAT values in the CKD population, as 29% of the patients did not have the 500 mg of iron stores included in their iron deficit when it was calculated using the modified Ganzoni formula. Overall, the average calculated iron deficit in patients from Studies 6 and 7 was 1392 mg.

Data from Study 7 reinforced the benefits of higher IV iron dosing such that significantly fewer patients who received a total cumulative dose of 1500 mg of iron required IV iron retreatment during the follow-up period (Days 56–90) than those who received a total cumulative dose of 1000 mg. In addition, patients who received 1500 mg of iron achieved their first Hb >11 g/dL and >12 g/dL and a ≥1 g increase in Hb faster than those who received 1000 mg. This finding suggests that patients given 1000 mg may not be receiving a full repletion dose of iron compared to those given 1500 mg. Study 6 was not similarly analyzed because of confounders (i.e., small sample size and lack of consistent dosing for comparators). Despite patients in Cohort D (IV SoC) of that study having the highest mean calculated iron deficit (1703 mg), the mean amount of iron they received was paradoxically only 812 mg. This discrepancy between deficit and treatment in patients who received IV SoC may be due, in part, to convenience factors associated with the IV SoC dosing available to the investigators during the study, as well as the lack of practical guidance for determining iron dosing requirements.

In a study that compared the Ganzoni calculated dose to a simplified dose regimen, it was found that adherence was higher with the simplified dosing and resulted in better efficacy outcomes [59]. As a result, standard of care in Europe has moved from the Ganzoni calculation to a simple dosing scheme. In the US, most of the IV iron has a simple dosing scheme and the Ganzoni formula is not utilized as frequently, our study suggests that the simplified dosing scheme most often utilized may not fully replete the iron stores of the majority of patients.

Although the results of our study suggest that a total cumulative dose of IV iron greater than 1000 mg may be appropriate for many patients with IDA (we are aware of no similar published analyses), there are some limitations to consider. Parts of our analyses were retrospective in nature, and further prospective research will be needed to establish the long-term efficacy and safety of these higher total cumulative doses of IV iron. The population analyzed from Study 7 was limited to patients with CKD. Other etiologies of IDA may respond differently in relation to IV iron. In addition, most of the studies that evaluated the Ganzoni formula included patients with IDA resulting from a variety of disease states. Also while, to the author's knowledge significant efficacy differences between similar cumulative doses of the various IV iron products have not been demonstrated, a future prospective study comparing various doses of the same product in a homogenous patient population would remove any product or population related bias that may have occurred in our study. It may be beneficial to observe whether higher or lower total cumulative doses of the same IV iron are more efficacious for patients with specific IDA etiologies.

## 5. Conclusions

Our study suggests that a total cumulative dose of 1000 mg of IV iron may be insufficient for iron repletion in the majority of patients with IDA and that a dose of 1500 mg is closer to the actual iron deficit in these patients. Additionally, 1500 mg of iron resulted in a more rapid, robust Hb response, allowed more patients to reach target Hb levels, and required a longer mean time to retreatment with additional IV iron compared to 1000 mg of iron. Our analysis and review of the literature suggest that 1500 mg of IV iron is more suitable for iron repletion in many patients with IDA compared to the commonly utilized dose of 1000 mg of IV iron. Further studies to confirm appropriate dose requirements in various patient populations are warranted.

## Figures and Tables

**Table 1:** Potential role of iron therapy in management of anemia [12].

Condition	Expected hepcidin levels	Iron parameters	Iron therapy strategies	Potential hepcidin therapy
Absolute iron deficiency anemia (IDA)	Low	Low TSAT and ferritin	PO or IV if poorly tolerated or malabsorbed	No

Functional iron deficiency (ESA therapy, CKD)	Variable, depending on ±CKD	Low TSAT, variable ferritin	IV	Antagonist (if hepcidin levels are not low)

Iron sequestration (anemia of inflammation (AI))	High	Low TSAT, normal-to-elevated ferritin	IV	Antagonist

Mixed anemia (AI/IDA or AI/functional iron deficiency)	Variable	Low TSAT, low-to-normal ferritin	IV^*∗*^	Antagonist (if hepcidin levels are not low)

TSAT = transferrin saturation; PO = oral; IV = intravenous; CKD = chronic kidney disease; ESA = erythropoiesis-stimulating agent.

^*∗*^Mixed anemia is a diagnosis of exclusion without a therapeutic trial of iron.

From [12].

**Table 2:** Current FDA-approved intravenous iron preparations [8, 10, 27–36].

Trade name	Dexferrum (iron dextran injection, USP)	INFeD (iron dextran injection, USP)	Ferrlecit (sodium ferric gluconate complex in sucrose injection)	Venofer (iron sucrose injection, USP)	Feraheme (ferumoxytol)	Injectafer (ferric carboxymaltose injection)
Manufacturer	American Regent, Inc.	Actavis Pharma, Inc.	Sanofi-Aventis	American Regent, Inc.	AMAG Pharmaceuticals	American Regent, Inc.

Test dose	Yes	Yes	No	No	No	No

Black box warning	Yes	Yes	No	No	Yes	No

FDA-approved indications	Iron deficiency where oral iron administration is unsatisfactory or impossible	Iron deficiency where oral iron administration is unsatisfactory or impossible	Iron deficiency anemia in adult and pediatric CKD patients receiving hemodialysis and receiving ESAs	IDA in adult and pediatric patients with non-dialysis-dependent, hemodialysis dependent, and peritoneal dialysis-dependent CKD	IDA in adult patients with CKD	IDA in adult patients who have intolerance to oral iron or have had unsatisfactory response to oral iron or adult patients with non-dialysis-dependent CKD

Total cumulative dose	Dependent on patient's total iron requirement	Dependent on patient's total iron requirement	1000 mg	1000 mg	1020 mg	1500 mg

CKD = chronic kidney disease; ESA = erythropoiesis-stimulating agent; IDA = iron deficiency anemia.

American Regent, Inc., is the human drug division of Luitpold Pharmaceuticals, Inc., Shirley, NY.

**Table 3:** Average calculated iron deficit dose in clinical Studies 1–5.

Study	Patient population	Calculated mean iron deficit based on the modified Ganzoni formula^*∗*^ (mg)	Standard deviation	Number of patients
(1) van Wyck et al., 2007 [38]	Postpartum	1458	330	182
(2) van Wyck et al., 2009 [39]	Heavy uterine bleeding	1608	383	251
(3) Seid et al., 2008 [40]	Postpartum	1539	351	143
(4) Barish et al., 2012 [41]	IDA various etiologies	1520	342	348
(5) Hussain et al., 2013 [42]	IDA various etiologies	1508^*∗∗*^	359	161
Overall mean		1531	NC	1085

IDA = iron deficiency anemia; NC = not calculated.

^*∗*^Patients randomized to receive IV iron based on a calculated iron deficit.

^*∗∗*^Including all randomized patients.

Data on file, Luitpold Pharmaceuticals, Inc.

**Table 4:** Average calculated iron deficit dose in clinical Studies 6 and 7.

Study	Patient population	Treatment group	Calculated mean iron deficit based on the modified Ganzoni formula (mg)	Standard deviation	Number of patients	Total mean
Study 6	IDA of various etiologies	Cohort 1 (A): 1500 mg IV iron Cohort 1 (B): oral iron Cohort 2 (C): 1500 mg IV iron Cohort 2 (D): IV SoC	1340 1344 1600 1703	356 360 446 482	246 253 252 245	1496 mg

Study 7 (REPAIR-IDA)	NDD-CKD	1500 mg IV iron 1000 mg IV iron	1355 1349	401 403	1275 1285	1352 mg

Overall mean			1392	NC	3556	

IDA = iron deficiency anemia; NDD-CKD = non-dialysis-dependent chronic kidney disease; SoC = standard of care; NC = not calculated.

Data on file, Luitpold Pharmaceuticals, Inc.

**Table 5:** Retreatment between Days 56–90 in clinical Study 7 (Safety Population).

	1500 mg IV iron (*n* = 1276)	1000 mg IV iron (*n* = 1285)	*p* value
*N* (%) patients retreated	71 (5.6%)	142 (11.1%)	*p* < 0.001

Data on file, Luitpold Pharmaceuticals, Inc.

**Table 6:** Hb >12 g/dL and end of treatment (Day 56) from clinical Study 7 (ITT population).

	1500 mg IV iron (*n* = 1249)	1000 mg IV iron (*n* = 1244)	*p* value
*N* (%) patients with Hb >12.0 g/dL	265 (24.4%)	169 (15.6%)	*p* = 0.001

Hb = hemoglobin; ITT = intent-to-treat.

Data on file, Luitpold Pharmaceuticals, Inc.

**Table 7:** Subjects with Hb >11 g/dL, 12 g/dL, or Hb change ≥1 g/dL in Study 7 anytime from randomization to end of study (Safety Population).

	1500 mg IV iron (*n* = 1276)	1000 mg IV iron (*n* = 1244)	Hazard ratio (95% CI)
*N* (%) of patients with Hb >11 g/dL	557 (56.1%)	504 (51.1%)	1.15 (1.02–1.30)
*N* (%) of patients with Hb >12 g/dL	358 (28.6%)	251 (20.0%)	1.44 (1.23–1.70)
*N* (%) of patients with Hb change ≥1 g/dL	610 (48.7%)	513 (41.0%)	1.27 (1.13–1.43)

Hb = hemoglobin.

Data on file, Luitpold Pharmaceuticals, Inc.
